# Evaluation of clinical factors and three scoring systems for predicting mortality in perforated peptic ulcer patients, a retrospective study

**DOI:** 10.1016/j.amsu.2021.102735

**Published:** 2021-08-21

**Authors:** M. Iqbal Rivai, Avit Suchitra, Aulia Janer

**Affiliations:** a Division of Digestive Surgery, Department of Surgery, Faculty of Medicine Andalas University - M.Djamil General Hospital, West Sumatera, 25171, Indonesia; b Department of Surgery, Faculty of Medicine Andalas University - M. Djamil General Hospital, West Sumatera, 25171, Indonesia

**Keywords:** ASA Classification, Boey's score, Mortality predictor, *Perforated peptic ulcer* (PPU), qSOFA score

## Abstract

**Background/objective:**

Early identification of mortality risk in perforated peptic ulcer (PPU) patients is important for triage and risk stratification. This study aimed to compare clinical and laboratory factors and three scoring systems to predict mortality in PPU patients.

**Methods:**

Retrospective data on PPU patients at M. Djamil Hospital who underwent emergency laparotomy repair surgery were collected from December 2018 to May 2021. The data included demographics, clinical characteristics, and three scoring systems. Data analysis used bivariate, multivariate, and ROC analysis.

**Results:**

A total 72 patients were included and mortality rate was 52.8%. Bivariate analysis showed a significant association between age (p = 0.029), onset of illness (p = 0.001), alteration of consciousness (p = <0.001), respiratory rate (p = 0.04), duration of surgery (p = 0.040), preoperative shock (p = 0.049), preoperative creatinine (p = <0.001), Boey's scores (p = 0.002), ASA (p = 0.001), and qSOFA scores (p = <0.001) with mortality in PPU patients. From multivariate analysis, the strongest clinical factors associated with mortality were alteration of consciousness (p = <0.001) and preoperative creatinine (p = 0.001). Receiver Operating Characteristic (ROC) analysis showed the area under the curve (AUC) of Boey's Score 0.73, ASA classification 0.69, qSOFA score 0.77, alteration of consciousness 0.74, and preoperative creatinine 0.78.

**Conclusion:**

Preoperative creatinine and altered consciousness had the strongest association with mortality in PPU patients. The qSOFA score predicted mortality better than Boey's score and ASA classification. Preoperative creatinine was the best single predictor of mortality.

## Highlights

• Perforated peptic ulcer is the severe complication with a high mortality rate
• Evaluation of the best mortality predictor in PPU patients is important
• Preoperative creatinine, alteration of consciousness were the strongest clinical factors as mortality predictor
• The accuracy of qSOFA score was superior to Boey's score and ASA classification
• Preoperative creatinine was the best single predictor of mortality.

## 1 Introduction

Peptic ulcer disease is an acid injury to the digestive tract, affecting the stomach and the proximal part of the duodenum. Currently, the most common causes are *H. pylori* infection and consumption of non-steroidal anti-inflammatory drugs (NSAIDs) [1]. Perforated peptic ulcer (PPU) is the severe complication with a mortality range 4–82.4% [2,3]. Many studies have been conducted to analyze the risk factors for PPU mortality. Risk factors that influence mortality are age, duration of surgery, delay in surgery, size of perforation, preoperative shock, high preoperative creatine, and albumin count. PULP score and Boey's score are widely used as scoring systems to predict PPU mortality [4,5], but their accuracy from various studies has varied. Another system used to predict mortality in PPU patients is ASA classification and qSOFA score.

Boey's score was a well-known scoring system for predicting mortality in PPU patients, consisting of three parameters: the presence of major illness, the onset of perforation >24 h, and preoperative shock [4]. The accuracy of Boey's score varies between studies, with some even suggesting that it is not superior. Currently, the PULP score has been introduced and included in WSES guidelines [6]. Still, its parameters such as the history of liver disease, AIDS, and active malignancy are challenging to identify in the study population due to lack of disease screening and poor patient education.

Early identification of mortality risk in PPU patients is important in triage and risk stratification. Knows which patients are at high risk of postoperative mortality is important in clinical decision-making, such as duration and degree of preoperative stabilization, and the appropriate timing of surgery. It is also essential for patients and families to make decisions. Therefore, this study aimed to compare clinical and laboratory factors and three scoring systems to predict mortality of PPU patients in our population.

## 2 Methods

### 2.1 Settings, ethics, and procedures

This retrospective cohort study was conducted at M. Djamil General Hospital, the tertiary care center in Padang, West Sumatera, Indonesia. The Health Research Ethics Committee of M. Djamil General Hospital has approved this study (#167/KEPK/2021). This study is registered in Researchregistry.com and written in accordance with the STROCSS statement [7]. All medical records of patients diagnosed with PPU and undergoing perforation repair laparotomy surgery from December 2018 to May 2021 were collected. Preoperative demographic and clinical data were collected, namely age, gender, preoperative shock, respiratory rate, alteration of consciousness, onset of illness, delay in surgery time, and patient comorbid status. Preoperative laboratory data such as preoperative creatinine and albumin levels were collected, as were the results of scoring systems such as the ASA classification, qSOFA, and Boey's score. Intraoperative data such as duration of surgery and size of perforation were included. Patients who were pregnant or had perforations of other gastrointestinal tracts were excluded.

### 2.2 Main outcome measure

The main outcome (primary endpoint) was after PPU repair surgery in-hospital mortality.

### 2.3 Definitions

Perforated peptic ulcer (PPU): includes gastric perforation and duodenal perforation.

The shock on admission: systolic blood pressure <90 mmHg and heart rate >100 beats per minute.

Respiratory rate (RR): increase in RR ≥ 22 times per minute [8].

Alteration of consciousness: determined by Glasgow coma scale (GCS) patient <15 [8].

The onset of illness: duration between the first time the patient suffered abdominal pain and admission to the hospital emergency department [4].

Delay in surgery: the duration between the patient's admission in the emergency department to the start of surgery.

Comorbidity: the presence of known comorbidities in PPU patients at the emergency department.

Boey's score: a score calculated by preoperative systolic blood pressure <100 mmHg, time from perforation onset to the emergency department admission >24 h, and presence of comorbidities [4].

ASA classification: subjective assessment performed by anesthesiologists to evaluate the risk of anesthetic procedures resulting in mortality [9].

qSOFA score: calculated by the presence of changes in mental status, respiratory rate >22 times per minute, and preoperative systolic blood pressure <100 mmHg [9].

### 2.4 Statistical analysis

Statistical analysis of the data in the study used SPSS version 25 (IBM Corp., Armonk, NY, USA). Population characteristics are described according to the type of variable. Bivariate analysis of each numerical variable on mortality using unpaired *t*-test and Mann Whitney test. Categorical variables used the chi-square test, Fisher's Exact, and Kruskal-Wallis. Variables with *p*-value < 0.2 from the bivariate analysis were then analyzed multivariate using binary logistic regression. All test results with p-value < 0.05 were accepted as statistically significant.

Receiver Operating Characteristic (ROC) analysis is a method to measure the predictor's ability to discriminate the subject outcome, disease or no disease. The optimal cut-off of each significant variable in multivariate analysis was calculated, including the three other scoring systems. Assessment of area under curve (AUC) also calculated by ROC analysis. AUC value > 0.90 is considered very excellent, while AUC 0.80–0.90 is considered excellent. AUC value of 0.7–0.8 is considered acceptable, AUC 0.50–0.70 is considered poor, and AUC <0.50 indicates no discrimination [10]. The accuracy of the significant variables from multivariate analysis as a single predictor was compared with the other three scoring systems, namely Boey's score, ASA score, and qSOFA score.

## 3 Results

Data from 72 patients who met the study criteria were analyzed. There were 43 male patients (59.7%), and 29 female patients (40.3%). The mean age of the patients was 64.8 (range 47–85) years, 38 patients deceased and the mortality rate was 52.8%. The demographics and characteristics of the patient population are summarized in Table 1. In the bivariate analysis, we found significant associations between several independent variables and mortality in PPU patients. The summary of the bivariate analysis is shown in Table 2 and Table 3.

**Table 1:** The demographics and characteristics of patient populations.

No.	Variable (n = 72)	n (%)	Mean ± SD	Median	Range
1.	Mortality				
	Alive	34(47.2)			
	Died	38(52.8)			
2.	Gender				
	Male	43(59.7)			
	Female	29(40.3)			
3.	Age (years)		64.8 ± 8,9	65.5	47–85
4.	Duration of surgery (hr)		2 ± 0.6	2	1–4.5
5.	Delay in surgery (hr)		15.9 ± 21	9	5–96
6.	Perforation Size		1.4 ± 0.9	1	0.5 - 4
7.	Preoperative Shock				
	Shock	8(11.1)			
	No Shock	64(88.9)			
8.	Preoperative Albumin		3 ± 0.5	3	2.2–4.3
9.	Preoperative Creatine		2.1 ± 1.1	1.9	0.4–4.4
10.	Onset of Illness (hr)				
	>24	63(87.5)			
	<24	9(12.5)			
11.	Increasing of Respiratory Rate				
	Yes	65(90.3)			
	No	7(9.7)			
12.	Alteration of Consciousness				
	Yes	23(31.9)			
	No	49(68.1)			
13.	Presence of Comorbidity				
	Yes	62(86.1)			
	No	10(13.9)			
14.	Boey's Score				
	Score 0	3(4.2)			
	Score 1	12(16.7)			
	Score 2	44(61.1)			
	Score 3	13(18.1)			
15.	ASA classification				
	ASA I	0(0)			
	ASA II	12(16.7)			
	ASA III	52(72.2)			
	ASA IV	8(11.1)			
	ASA V	0(0)			
16.	qSOFA				
	Score 0	5(6.9)			
	Score 1	43(59.7)			
	Score 2	16(22.2)			
	Score 3	8(11.1)			

SD= Standard Deviation.

**Table 2:** Bivariate analysis between Categorical Variables and Mortality.

Variables	Alive	Deceased	*p value*
	n (%)	n (%)	
Gender			
Male	19(44.2)	24(55.8)	0.53^a^
Female	15(51.7)	14(48.3)	
Preoperative shock			
Shock	1(12.5)	7(87.5)	0.049^b^
No Shock	33(51.6)	31(48.4)	
Onset of Illness (hr)			
>24	25(39.7)	38(60.3)	0.001^b^
<24	9(100)	0(0)	
Increasing of Respiratory Rate			
Yes	28(43.1)	37(56.9)	0.04^a^
No	6(85.7)	1(14.3)	
Alteration of Consciousness (GCS <15)			
Yes	2(8.7)	21(91.3)	<0.001^a^
No	32(65.3)	17(34.7)	
Presence of Comorbidity			
Yes	27(43.5)	35(56.5)	0.12^a^
No	7(70)	3(30)	
Boey's Score			
Score 0	3(100)	0(0)	0.002^c^
Score 1	10(83.3)	2(16.7)	
Score 2	19(43.2)	25(56.8)	
Score 3	2(15.4)	11(84.6)	
ASA			
ASA I	0(0)	0(0)	0.001^c^
ASA II	10(83.3)	2(16.7)	
ASA III	24(46.1)	28(53.9)	
ASA IV	0(0)	8(100)	
ASA V	0(0)	0(0)	
qSOFA			
Score 0	5(100)	0(0)	<0.001^c^
Score 1	26(60.4)	17(39.6)	
Score 2	3(18.7)	13(81.3)	
Score 3	0(0)	8(100)	

SD= Standard Deviation, ^a^ Chi square test, ^b^ Fisher's exact test, ^c^ Kruskal-wallis test.

**Table 3:** Bivariate analysis between Numerical Variables and Mortality.

Variables	Alive	Deceased	*p value*
	Mean ± SD	Mean ± SD	
Age (years)	62.44 **±** 7.7	66.97 **±** 9.3	0.02^a^
Duration of Surgery (Hours)	1.85 **±** 0.4	2.17 **±** 0.7	0.04^b^
Delay in Surgery (Hours)	14.18 **±** 21.08	17.57 **±** 21.01	0.05^b^
Perforation size (cm)	1.19 **±** 0.74	1.50 **±** 0.97	0.16^b^
Preoperative Albumin (g/dL)	3.09 **±** 0.55	2.97 **±** 0.52	0.38^b^
Preoperative Creatinine (mg/dL)	1.61 **±** 0.96	2.56 **±** 0.96	<0.001^b^

SD= Standard Deviation, ^a^ Independent *t*-test, ^b^ Mann-whitney test.

**Fig. 1:**
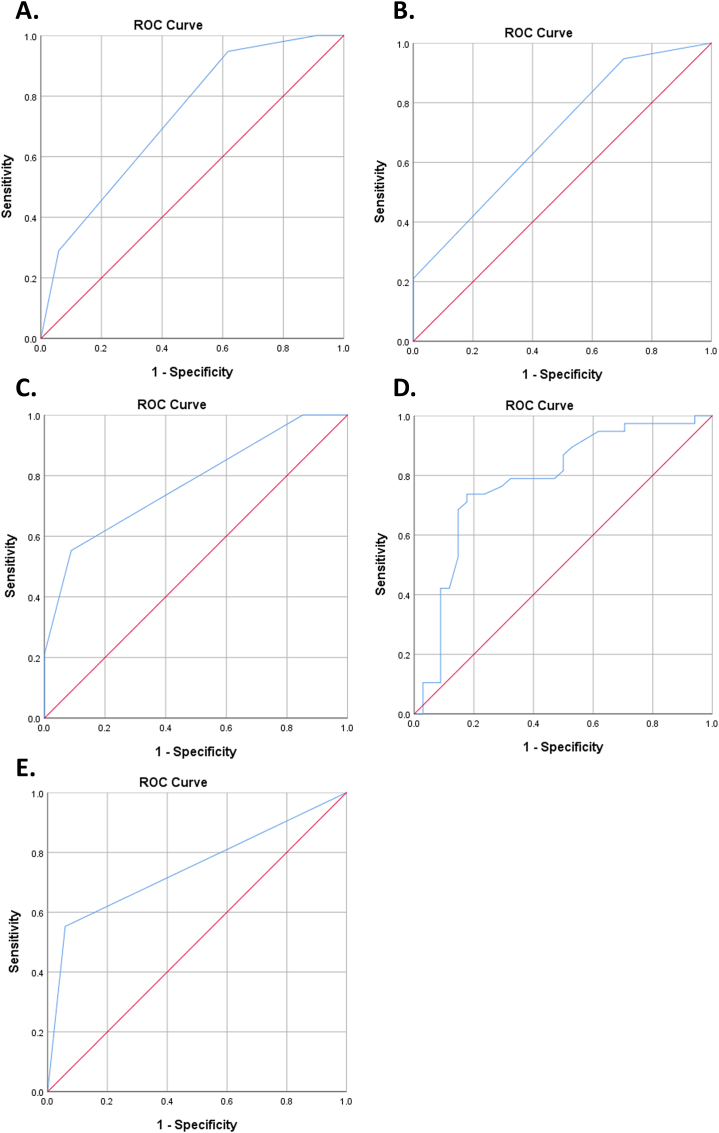
ROC Curve of Boey's Score (A), ASA Classification (B), qSOFA Score (C), Preoperative Creatinine (D), and Alteration of Consciousness (E).

In the multivariate analysis, we analyzed several preoperative clinical factors that had p < 0.2 in the bivariate analysis, and scores were not included in the analysis. Non-significant variables were taken out for optimization. The final result showed preoperative creatinine and alteration of consciousness were the strongest predictors (p = 0.001 and p=<0.001). The summary of the multivariate analysis was shown in Table 4. The optimal cut-off of the two strongest variables and the three scoring systems were calculated to classify the mortality risk. The accuracy of preoperative creatinine and altered consciousness in predicting risk of mortality was evaluated by ROC analysis, including three other scoring systems. ROC analysis is shown in Table 5, and the AUC is shown in Fig. 1.

**Table 4:** Summary of multivariate analysis for mortality.

No.	Variable	*p value*	OR	(95% CI)
1.	Preoperative Creatinine	0.001	2.89	(1.50–5.54)
2.	Alteration of Consciousness	<0.001	21.87	(4.08–117.09)

OR= *Odds Ratio*, CI= *Confidence Interval*.

**Table 5:** Optimal cut-off and diagnostic test.

Predictor	*Cut off*	AUC	Sensitivity (%)	Specificity (%)	PPV (%)	NPV (%)
Preoperative Creatinine (mg/dL)	≥2	0.78	73.68	76.47	77.77	72.22
Alteration of Consciousness	<15	0.74	55.26	94.11	91.30	65.30
qSOFA Score	≥2	0.77	55.26	91.17	87.50	64.58
Boey's Score	≥2	0.73	94.73	38.23	63.15	86.66
ASA	≥3	0.69	94.73	29.41	60	83.33

AUC = *Area Under Curve,* PPV= *Positive Predictive Value,* NPV= *Negative Predictive Value*.

## 4 Discussion

In this study, demographic, clinical characteristics, and existing scoring systems were analyzed. Bivariate analysis showed that age, duration of surgery, delay in surgery, preoperative creatinine, preoperative shock, onset of illness, RR, and altered consciousness had a statistically significant relationship with mortality of PPU patients. Each of the three scoring systems was also analyzed by bivariate analysis. Boey's score, ASA classification, and qSOFA score showed statistically significant associations with mortality in PPU patients. In multivariate analysis, we only analyzed preoperative clinical and laboratory factors to predict preoperative mortality. The strongest preoperative factors associated with PPU mortality were preoperative creatinine (p = 0.001) and alteration of consciousness (p = <0.001). Multivariate analysis by Thorsen et al. found age, delay in surgery, active cancer, preoperative albumin, creatinine, and bilirubin were the strongest associated variables (p = 0.001, p = 0.03, p = 0.005, p = 0.01, p = 0.01, and p = 0.03) [11]. Researchers from Indonesia, Smaradhania et al. found onset >24 h, preoperative shock and preoperative creatinine were the strongest predictors (p = 0.02, p = 0.01, p = 0.02) [12]. Lee et al. also used multivariate analysis and obtained Boey's score significantly associated with PPU mortality [13].

In this study, preoperative creatinine had a strong association with PPU mortality. Two articles have shown an increase in creatinine serum associated with in-hospital and 30-day post-surgical mortality [14,15]. ROC analysis was performed to evaluate its accuracy, the AUC value of preoperative creatinine was 0.78 as acceptable discrimination. Based on the values of sensitivity, specificity, PPV, and NPV, preoperative creatinine predicts mortality in PPU patients better. Thorsen et al. also analyzed the preoperative creatinine AUC value, and the AUC result was 0.52 as poor discrimination. They reported albumin was the strongest single predictor with an AUC value of 0.78, contrary we did not find similar result. Creatinine represents renal function indirectly, the KIDIGO classification defines acute kidney injury (AKI) as occurring when serum creatinine rises by 0.3 mg/dl or more from baseline for 24 h [16]. The main etiology of AKI is renal hypoperfusion which is caused by dehydration, blood loss, and cardiogenic shock. In sepsis, AKI occurs due to concomitant systemic hypoperfusion and intrarenal vasodilation [17]. Renal failure was one of the parameters in the SOFA score to predict sepsis [8]. We found no history of chronic renal failure or history of renal replacement therapy in these subjects, therefore the increase in serum creatinine is most likely due to dehydration and sepsis.

Alteration of consciousness also had a strong association with PPU mortality. We have not found another multivariate study that specifically analyzed the association between altered consciousness and PPU mortality. However, Uzman et al. conducted a study of surgical patients in the intensive care unit and found patients with low GCS scores had higher mortality rates [18]. Furthermore, GCS is one of the components in the APACHE II score and POSUM score [19,20]. Changes in consciousness or decreased GCS score in patients reflect decreased brain function that is strongly correlated with sepsis. In septic conditions, brain injury occurs due to impaired cerebral microcirculation, altered neurotransmission, endothelial and blood-brain barrier (BBB) dysfunction, and inflammatory mediator together with complement system can injure the BBB and endothelial layer [21]. Sprung et al. reported that the mortality rate of septic patients with altered mental status was 49%, and the mortality rate of septic patients without neurological symptoms was 26% [22]. The AUC value of alteration of consciousness was 0.74 as acceptable discrimination, but the sensitivity was not higher than preoperative creatinine (55.26%).

This study compared three clinical scoring systems to predict the mortality of PPU patients. We analyzed these scores because their parameters are easy to obtain in our hospital's emergency department. ROC analysis and diagnostic test performed on Boey's score, ASA, qSOFA score. The qSOFA had a better accuracy than two others to predict mortality in PPU patients with an AUC value was 0.77 as acceptable discrimination. The AUC value of Boey's score was 0.73, and the ASA classification was 0.69. The qSOFA is a simple scoring system representing sepsis and organ failure risk published by the European Society of Intensive Care Medicine (ESICM) in 2016 [8]. The qSOFA consists of three parameters and assigns one score to each parameter if blood pressure <100 mmHg, respiratory rate >22 breaths per minute, and decreased level of consciousness (GCS <15). Patients with a qSOFA score of 2 or more have a higher risk of death and a longer stay in the intensive care unit [9]. Our subject mostly deceased within the septic condition, in our bivariate result, all components of qSOFA were statistically significant with PPU mortality. Similarly, Uwais et al. report qSOFA had a significant association with mortality in PPU patients [3]. We found no other study that specifically evaluated the accuracy of the qSOFA score for predicting mortality in PPU patients. Lo et al. evaluated the accuracy of the qSOFA score for predicting mortality in general, with an AUC of 0.68 as poor discrimination, contradicting our result [23]. Although Boey's score has long been known and used, some current studies showed its accuracy was unsatisfactory. Buck et al. showed the AUC value of Boey's score was 0.63 as poor discrimination [21], and Saafan et al. reported AUC of Boey's score was 0.69 also as poor discrimination [13]. This study showed that the Boey's score had a high false-positive with the specificity value was only 38.23% compared to the qSOFA score.

Another scoring system used to predict mortality in PPU patients was ASA classification. It is a subjective assessment by the anesthesiologist that consists of five categories, namely ASA I – V. This score assesses the patient's condition before surgery, used to evaluate the risk of mortality in anesthetic procedures [24]. In this study, the AUC of the ASA classification was 0.69 as poor discrimination. The specificity of the ASA classification is low 29.41%, which means high false positives. This result is different from other studies, Buck et al. found that the ASA classification has acceptable discrimination with an AUC of 0.73. Thorsen et al. also found the same result where the AUC value was 0.79. Due to the subjectivity of the ASA assessment by the anesthesiologist, applying the ASA classification as a predictor of mortality should be considered wisely.

The mortality rate of PPU patients in this study was 52.8%, and worldwide mortality rates vary. The lowest mortality rate was 4% and the highest mortality rate was 82.4% [2,3]. The high mortality rate in this study was strongly associated with preoperative creatinine levels and alteration of consciousness. These two clinical factors represent multi-organ failure (kidney and brain). So, we conclude, the condition of the PPU patients when they were admitted to our hospital was late and in a state of severe sepsis. There was a social phenomenon in our region in which patients preferred to delay seeking health care to the hospital. Most patients try to self-medicate with over-the-counter medications, such as pain relievers, antacids, and even antibiotics. Due to cultural beliefs, patients also often seek traditional or alternative medicine first. This condition has also been observed by Widayanti et al. [25].

We estimate that similar conditions also occurred in Uwais et al.'s study population, located in Samarinda, East Kalimantan (82.4% mortality). We hope that this research can be a reference for clinicians and the government to improve education and health promotion for the community. This study has limitations; the data from this study were obtained retrospectively. In the future, a more extensive study population may reveal relationships not seen in this study.

## 5 Conclusion

Preoperative creatinine and altered consciousness had the strongest association with mortality in PPU patients. The qSOFA score predicted mortality better than Boey's score and ASA classification. Preoperative creatinine was the best single predictor of mortality.

## Declaration of competing interest

None.

## Trial registry number

1. Name of the registry: Researchregistry.com
2. Unique Identifying number or registration ID: researchregistry6975
3. Hyperlink to your specific registration (must be publicly accessible and will be checked): https://www.researchregistry.com/browse-the-registry#home/registrationdetails/60f2c84ffdca39001ef9a933/
